# Biomimetic Modular Honeycomb with Enhanced Crushing Strength and Flexible Customizability

**DOI:** 10.3390/ma17204950

**Published:** 2024-10-10

**Authors:** Lumin Shen, Yuanzhi Wu, Tuo Ye, Tianyu Gao, Shanmei Zheng, Zhihao Long, Xi Ren, Huangyou Zhang, Junwen Huang, Kai Liu

**Affiliations:** 1 College of Intelligent Manufacturing and Mechanical Engineering, Hunan Institute of Technology, Hengyang 421002, China; 2015001995@hnit.edu.cn (L.S.); 2013001767@hnit.edu.cn (Y.W.); 2017001002@hnit.edu.cn (T.Y.); 17873627570@163.com (S.Z.); 15570925214@139.com (Z.L.); rx773333@163.com (X.R.); zhy040601@126.com (H.Z.); 15671776199@163.com (J.H.); 2 School of Traffic & Transportation Engineering, Central South University, Changsha 410075, China; 204207030@csu.edu.cn

**Keywords:** biomimetic, honeycomb, enhanced crushing strength, flexible customizability

## Abstract

The integration of biomimetic principles into the sophisticated design of honeycomb structures has gained significant traction. Inspired by the natural reinforcement mechanisms observed in tree stems, this research introduces localized thickening to the conventional honeycombs, leading to the development of variable-density honeycomb blocks. These blocks are strategically configured to form modular honeycombs. Initially, the methodology for calculating the relative density of the new design is meticulously detailed. Following this, a numerical model based on the plastic limit theorem, verified experimentally, is used to investigate the in-plane deformation models of modular honeycomb under the low- and high-velocity impact and to establish a theoretical framework for compressive strength. The results confirm that the theoretical predictions for crushing strength in the modular honeycomb align closely with numerical findings across both low- and high-velocity impacts. Further investigation into densification strain, energy absorption, and gradient strategy is conducted using both simulation and experimental approaches. The outcomes indicate that the innovative design outperforms conventional honeycombs by significantly enhancing the crushing strength under low-velocity impacts through the judicious arrangement of honeycomb blocks. Additionally, with a negligible difference in densification strains, the modular honeycomb demonstrates superior energy dissipation capabilities compared to its conventional counterparts. At a strain of 0.85, the modular honeycomb’s energy absorption capacity improves by 36.68% at 1 m/s and 25.47% at 10 m/s compared to the conventional honeycomb. By meticulously engineering the arrangement of sub-honeycombs, it is possible to develop a modular honeycomb that exhibits a multi-plateau stress response under uniaxial and biaxial compression. These advancements are particularly beneficial to the development of auto crash absorption systems, high-end product transportation packaging, and personalized protective gear.

## 1. Introduction

The honeycomb structure is a typical example of cellular material, exhibiting a two-dimensional hexagonal cell that extends in-plane and a parallel stacking configuration in the out-plane direction [1,2]. The interconnection of the unit cells efficiently supports the spatial configuration of the structure, endowing honeycombs with high porosity and low mass relative to the constituent material [3]. As a consequence, honeycombs are characterized by superior specific stiffness, specific strength, and high energy absorption capacity [4].

Leveraging the lightweight and high energy absorption efficiency of honeycomb, it finds extensive applications across various engineering fields, particularly where efficient energy dissipation is critical [5,6,7,8]. Researchers delve into the study of honeycomb structures, emulating their natural topological configurations. Utilizing materials such as aluminum, paper, and plastic as substrates, components with honeycomb-like geometries are crafted to meet diverse engineering requirements. In the transportation sector, honeycomb structures are employed in the fabrication of energy-absorbing crumple zones, designed to absorb and disperse impact forces during collisions, thereby safeguarding occupant safety [9,10,11,12]. In aerospace applications, these structures are favored for their lightweight and high-strength attributes, finding use in aircraft wing ribs and satellite structural components, where weight reduction and enhanced load-bearing capacity are paramount [13,14,15,16,17]. In the packaging field, honeycomb structures serve as cushioning materials, protecting goods from the shocks and vibrations inherent in transit [18,19,20,21,22]. In military defense, honeycombs are incorporated into the design of body armor and armored vehicles to withstand high-velocity impacts [23,24,25,26]. When subjected to impact, these honeycomb structures demonstrate a prolonged plateau stress phase, allowing the material to sustain significant forces over an extended period. This characteristic facilitates the gradual conversion of kinetic energy into plastic deformation energy of the material, effectively absorbing and dispersing impact forces and safeguarding the structural integrity [27,28,29].

Increasingly demanding engineering scenarios pose higher requirements for the development of materials [30,31]. In the field of lightweight energy-absorbing materials, improving the specific energy absorption of honeycombs is a continuous pursuit [32,33,34,35,36,37]. Accordingly, a substantial amount of honeycomb designs were proposed, such as gradient honeycombs composed of layers of cells with varying densities [38,39,40,41], vertex-based hierarchical honeycombs that replace the vertices with self-similar structures [37,42,43], honeycombs filled with other structures in the cells [44,45], and cell-walled hierarchical honeycombs with walls replaced by other porous structures [2,46]. These new configurations provide enhanced energy dissipation capabilities and structural advantages, partially catering to the demands of advanced applications where traditional honeycomb designs may fall short. Furthermore, as demands for the intelligent adaptability of energy-absorbing structures escalate, particularly in applications such as sports equipment and packaging materials, the design of customizable plateaus stress for energy absorbers has become an important issue that urgently needs to be addressed [47]. This presents a challenge due to the intricate nonlinearity inherent in the relationship between structural geometry and mechanical performance. Several studies have delved into responses to these challenges [48,49,50,51,52]. Wang et al. [53] introduced a self-similar structure within a face-centered cubic lattice, achieving a customizable plateau stress. Yu et al. [54] proposed a novel heterogeneous strategy, integrated with artificial neural networks, to tailor the mechanical behavior of structures to specific requirements. These studies provide insights into the customized design of the plateau stress for porous materials.

However, existing research seldom design honeycomb topologies capable of simultaneously enhancing both load-bearing capacity and energy absorption, while also enabling the customization of the plateau stress. For instance, Zhang et al. [55] proposed a hierarchical honeycomb resembling a spider web, which enhanced its mechanical properties, but the customizable properties are not described. Similarly, Tao et al. [56] constructed six hierarchical honeycombs by replacing each vertex of regular hexagonal and square honeycombs with three small-scale configurations, which strengthened the honeycomb’s mechanical strength, yet did not achieve the customization of the mechanical properties. On the other hand, Li et al. [57] designed a honeycomb with adjustable mechanical properties, but there is a lack of research on the mechanical enhancement of the designed honeycomb. Drawing inspiration from the biomimetic idea of variable density, this study designs honeycomb blocks with local various wall thicknesses. It is well recognized that trees in the natural world display a variable density gradient, with the trunk’s central region having a substantially higher tissue density compared to the surrounding areas. This density variation bestows trees with exceptional mechanical properties, such as increased resistance to impact forces. By strategically arranging honeycomb blocks with varying densities following specific patterns, it becomes feasible to design honeycombs that can tailor the plateau stress to specific requirements.

Differing from previous studies, this study adjusts the wall thickness of the honeycomb locally to enhance the specific energy absorption and arranges modular honeycombs strategically to customize the plateau stress. For the proposed biomimetic design, numerical and theoretical methods are used to explore the in-plane impact deformation behavior of the modular honeycomb and its enhanced mechanical performance mechanism. The study investigated the effects of varying density distributions on the densification strain and energy absorption capability of modular honeycombs with equal mass, ultimately providing a strategy for customizing the plateau stress. Overall, the deformation mode, the enhanced impact resistance mechanism, and the plateau stress customization strategy are successively investigated to determine the advantages of the new design, paving the way to applications in the fields where energy absorption and intelligent adaptability are paramount.

## 2. Modular Biomimetic Honeycomb Design

### 2.1. Geometric Model

**Figure 1:**
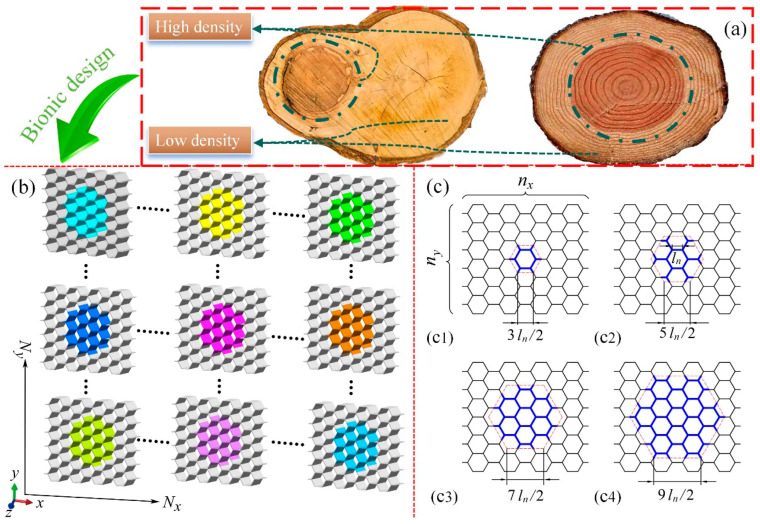
The design inspiration and geometry of the modular honeycomb. (**a**) The phenomenon of variable density in tree trunks, (**b**) the design process of modular honeycomb, and (**c**) four distinct configurations of the various density honeycomb blocks, (**c1**) The honeycomb block with side length 3*l_n_*/2, (**c2**) The honeycomb block with side length 5*l_n_*/2, (**c3**) The honeycomb block with side length 7*l_n_*/2, (**c4**) The honeycomb block with side length 9*l_n_*/2.

As illustrated in Figure 1a, tree trunks display a variation in density, with the center of the trunk being denser than the adjacent tissues. This phenomenon endows trees with superior mechanical properties, including enhanced resistance to toppling and impact. Inspired by the density variation in tree trunks, which provides them with exceptional mechanical strength and impact resistance, this study introduces a biomimetic variable density approach to the design of honeycomb blocks to mimic the efficient structural reinforcement seen in nature.

Figure 1b presents that the modular honeycomb consists of $N_{x}$ and $N_{y}$ blocks along the *x*- and *y*- directions, respectively. Each block comprises a centrally located, colored, thick-walled sub-honeycomb, which is enveloped by a thin-walled matrix honeycomb. Figure 1c illustrates four distinct configurations of the various density honeycomb blocks, with each sub-honeycomb featuring a regular hexagonal outline. The side lengths of these hexagons are multiples of the cell edge length, such as 1.5, 2.5, 3.5, and 4.5 times, with the maximum length of the sub-honeycomb outline being constrained by the overall length of the honeycomb blocks. The honeycomb blocks contain $n_{x}$ cells along the *x*-axis and $n_{y}$ cells along the *y*-axis, where $n_{x}$ corresponds to the number of complete hexagons in the *x*-direction, and $n_{y}$ corresponds to the count of complete hexagons in the *y*-direction. As depicted in Figure 1c1, $n_{x}$ and $n_{y}$ are 4 and 6, respectively. For ease of reference, the three honeycomb block arrangements in Figure 1c2 to Figure 1c4 are designated as BJ1, BJ2, and BJ3, respectively.

**Figure 2:**
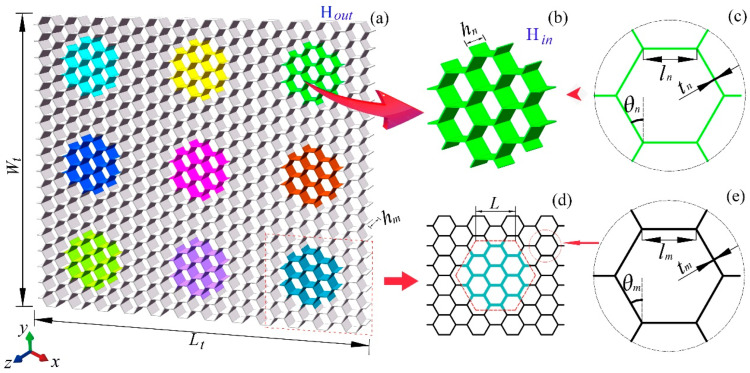
The 3 × 3 modular honeycomb structure (**a**), sub-honeycomb configuration (**b**) and geometric parameters (**c**), and matrix honeycomb configuration (**d**) and geometric parameters (**e**).

$N_{x}$ and $N_{y}$ affect the total absorbed energy and load capacity of the modular honeycomb. However, different configurations of the modular honeycomb share the same impact enhancement mechanism. Without loss of generality, this work focuses on a 3 × 3 modular honeycomb for impact resistance enhancement research, as shown in Figure 2a. Figure 2b,c define the geometric parameters of the sub-honeycomb cell, including the cell wall length $l_{n}$, the angle between the cell wall and the vertical direction $\theta_{n}$, the cell wall thickness $t_{n}$, and the height of the honeycomb wall in the *z*-direction $h_{n}$. Figure 2d,e display the geometric parameters of the matrix honeycomb cell $l_{m}$, $\theta_{m}$, and $t_{m}$. Considering the predominance of regular hexagonal honeycombs in engineering applications, $\theta_{m}$ and $\theta_{n}$ are set to 30°. The cell wall lengths and heights of the sub-honeycomb and matrix honeycomb are equal, i.e., $l_{n}=l_{m}$, $h_{n}=h_{m}$, where $h_{m}$ represents the height of the matrix honeycomb along the *z*-axis. After these specifications for the geometric parameters of the modular honeycomb, its mechanical behavior is primarily modified through the alteration of the wall thickness within the sub-honeycomb and matrix honeycomb, coupled with the strategic arrangement of the honeycomb blocks.

### 2.2. Relative Density

The relative density $\rho_{r}$ is important for porous materials, representing the proportion of solid material in the honeycomb structure. It is defined as

(1) $$\rho_{r}=\frac{\rho_{e}}{\rho_{0}}$$

in which, $\rho_{e}$ represents the effective density, while $\rho_{0}$ denotes the density of the base material.

**Figure 3:**
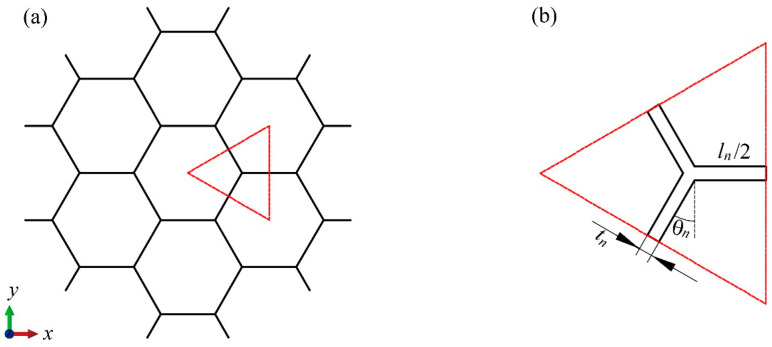
A sub-honeycomb (**a**) and the typical repetitive unit of the honeycomb structure (**b**).

We use the BJ2 honeycomb block as an example to derive the formula for the relative density of the modular honeycomb. As illustrated in Figure 3a, the sub-honeycomb comprises 24 basic elements, each delineated by a triangular frame. Figure 3b provides a detailed view of the basic elements, along with its geometric parameters. The mass of the sub-honeycomb is equivalent to the product of the total volume of all cell walls and the density of the base material. Therefore, the total mass $m_{n}\;$of the sub-honeycomb is deduced as

(2) $$m_{n}=36l_{n}t_{n}h_{n}\rho_{0}$$

The BJ2 block comprises 72 basic elements in the matrix honeycomb, hence the mass of the matrix honeycomb is

(3) $$m_{m}=108l_{m}t_{m}h_{m}\rho_{0}$$

The effective density of the BJ2 block $\;\rho_{e}$, which is equal to the mass of the block divided by the volume, is

(4) $$\rho_{e}=\frac{9m_{m}+\sum_{n=1}^{9}m_{n}}{648\sqrt{3}{l_{m}}^{2}h_{m}}$$

Therefore, the relative density of the BJ2 block is

(5) $$\rho_{r}=\frac{27\sqrt{3}t_{m}h_{m}+\sqrt{3}\sum_{n=1}^{9}t_{n}h_{n}}{54l_{m}h_{m}}$$

The relative density calculation formula for a modular honeycomb, configured with $N_{x}$ and $N_{y}$ honeycomb blocks along the *x*- and *y*-directions, respectively, and containing $n_{x}$ and $n_{y}$ cells within a single block in the horizontal and vertical directions, respectively, while keeping the count of sub-honeycomb cell walls constant, is expressed as follows:

(6) $$\rho_{r}=\frac{{\sqrt{3}N}_{x}N_{y}\left(2n_{x}n_{y}-12\right)t_{m}h_{m}+12\sqrt{3}\sum_{n=1}^{N_{x}N_{y}}t_{n}h_{n}}{{3N}_{x}N_{y}n_{x}n_{y}l_{m}h_{m}}$$

## 3. Finite Element Models

**Figure 4:**
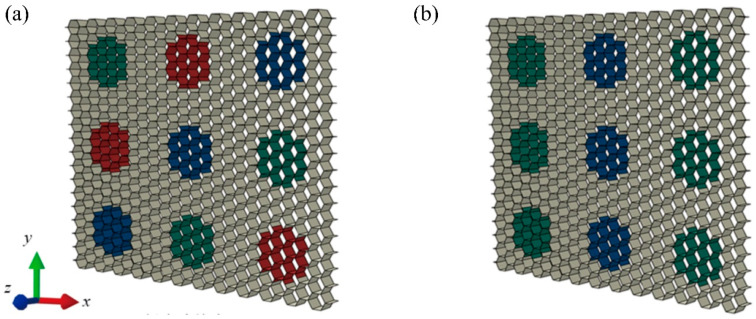
Two different types of 3 × 3 modular honeycombs. (**a**) The modular honeycomb with randomly distributed honeycomb blocks, (**b**) The modular honeycomb neat honeycomb blocks.

Based on the configurational rule of modular honeycombs, a diverse array of such structures can be crafted by tuning the arrangement of honeycomb blocks and modulating the wall thickness of the sub-honeycombs. Figure 4 illustrates two types of modular honeycombs, with the sub-honeycombs displayed in different colors to represent varying wall thicknesses. Despite the variances in these structural configurations, the mechanisms by which these modular honeycombs enhance impact resistance and how the wall thickness of sub-honeycombs influences the deformation modes are consistent across configurations. This section specifically examines the modular honeycomb shown in Figure 4b to delineate the methodology for constructing an in-plane compression numerical model tailored to modular honeycomb structures.

**Figure 5:**
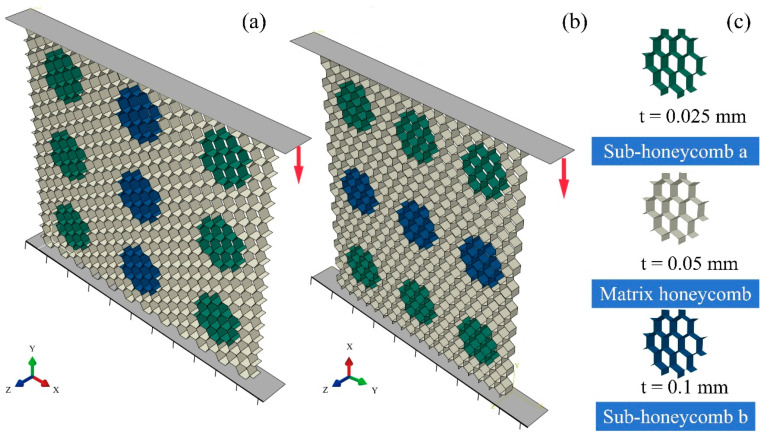
The numerical model of in-plane compression for the modular honeycomb. *y*-directional compression (**a**), *x*-directional compression (**b**), wall thickness of the sub-honeycomb and matrix honeycomb (**c**).

To gain a comprehensive understanding of the deformation patterns and the mechanical characteristics of modular honeycombs, a finite element analysis was conducted utilizing Abaqus/CAE|SIMULIA 2023. Figure 5a,b represent the numerical models of the modular honeycomb subjected to compression along the *y*- and *x*-directions, respectively. In Figure 5c, the dark green, metallic, and blue honeycombs represent the sub-honeycomb a, matrix honeycomb, and sub-honeycomb b, respectively, with thicknesses of 0.025 mm, 0.05 mm, and 0.1 mm.

The structure is sandwiched between a pair of rigid walls. The upper wall presses down at a uniform velocity, while the lower one remains stationary. Restriction on the *z*-directional displacement of the honeycomb is implemented to avert any tilting during the compression process. Both the matrix honeycomb and the sub-honeycombs of various types are regular hexagons, each with a uniform edge length of 4 mm and a height of 1 mm. Considering the thin-walled characteristics of the structure, the structure is discretized with S4R elements. The coefficient of friction, which applies to interactions between the honeycomb and the rigid walls as well as internally within the honeycomb, is uniformly established at 0.15. Aluminum is selected as the base material for the honeycomb, with a density of 2700 kg/m^3^, an elastic modulus of 68.2 GPa, and a Poisson’s ratio of 0.3. To account for the strain-hardening behavior of aluminum, the stress–strain data presented in Table 1 are incorporated into the model.

**Figure 6:**
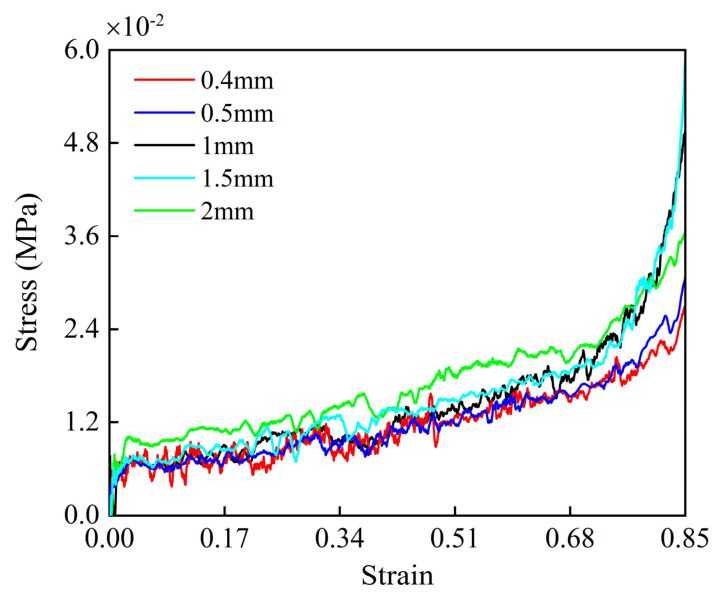
The mesh convergence study of the numerical model.

To balance the trade-off between computational time and accuracy, a mesh size convergence study was conducted for the loading condition in the *y*-direction. Figure 6 displays the stress–strain curves derived from simulations utilizing different mesh sizes at a loading velocity of 1 m/s. The stress–strain curves for the 0.4 mm and 0.5 mm meshes are close, suggesting that the finite element analysis reached convergence with a mesh size of 0.5 mm. Therefore, the 0.5 mm mesh size is selected for the subsequent computational simulations.

**Figure 7:**
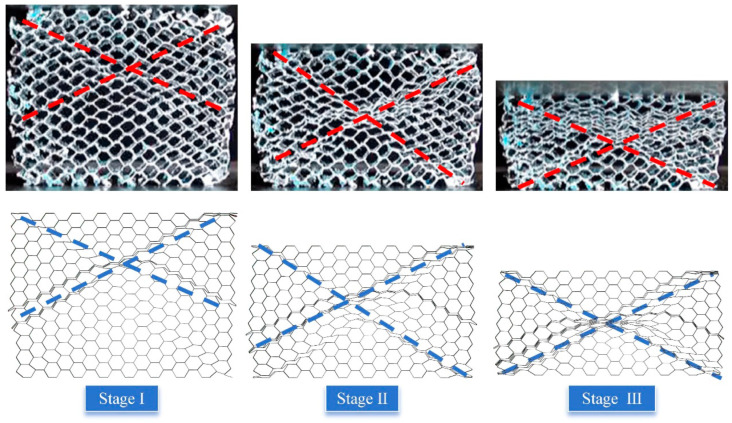
The verification of numerical methods through comparative experiment.

The study [59] performed in-plane compression tests on a conventional aluminum honeycomb and elucidated the deformation behavior when subjected to *y*-direction compression. To substantiate the reliability of the numerical method, a computational model corresponding to the experiment in the reference was constructed using the numerical method. The geometric and material parameters are consistent with those in the experiment, while others are the same as in the simulation method. Figure 7 juxtaposes the deformation modes observed during *y*-direction compression in both the experiment and the numerical simulation. The numerically predicted deformation modes at various strain levels closely match the experimental observations, particularly the formation of the X-shaped bands within the honeycombs, which demonstrates the reliability of the numerical model.

## 4. In-Plane Dynamic Crushing Behaviors

This section utilizes numerical simulation to construct models for the modular honeycomb subjected to low-velocity and high-velocity impacts. The outcomes of these simulations are then employed to explore the deformation modes of the modular honeycomb in both the *x*- and *y*-directions.

### 4.1. Low-Velocity Crushing

#### 4.1.1. Deformation Mode under Low-Velocity Crushing

**Figure 8:**
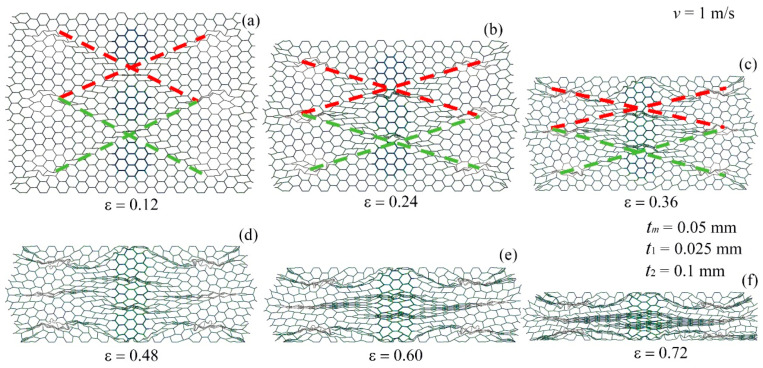
The deformation mode of the modular honeycomb when subjected to *y*-direction compression at the velocity of 1 m/s. (**a**) ε=0.12, (**b**) ε=0.24, (**c**) ε=0.36, (**d**) ε=0.48, (**e**) ε=0.60, (**f**) ε=0.72.

The deformation modes of the modular honeycomb during *y*-directional low-velocity compression are depicted in Figure 8. The thin-walled sub-honeycombs deform before the thick-walled counterparts. Upon reaching a strain of approximately 0.12, the thin-walled sub-honeycombs experience significant plastic deformation. At this stage, the modular honeycomb exhibits two vertically aligned X-shaped deformation bands along the lines connecting the thin-walled sub-honeycombs, while the thick-walled sub-honeycombs remain largely undeformed.

As the strain increases to 0.24, the thick-walled sub-honeycombs, supported by their robust cell walls, along with the matrix honeycombs interspersed with them, continue to retain a relatively intact hexagonal configuration. Conversely, the matrix honeycombs positioned between two thick-walled sub-honeycombs undergo collapse. Concurrently, the previously formed X-shaped deformation bands within the modular honeycomb are compressed further, becoming flatter because of the increased strain. As the strain increases to 0.36, the deformation of the thick-walled sub-honeycombs becomes gradually pronounced, while the thin-walled sub-honeycombs, which are originally at the same horizontal position, become densified. The local support effect formed by the thick-walled sub-honeycombs makes it difficult for the matrix honeycombs, which are misaligned with them, to deform, effectively taking the place of the originally thin-walled sub-honeycombs and aligning horizontally with the thick-walled sub-honeycombs. The X-shaped deformation band formed in the modular honeycomb becomes increasingly compressed.

At a strain of 0.48, the thick-walled sub-honeycombs experience substantial plastic deformation, with the X-shaped deformation bands becoming compacted. This compaction results in the formation of three I-shaped deformation bands, characterized by a “bulge” at the center, reminiscent of the character “Φ”. As the strain approaches approximately 0.60, the thin-walled sub-honeycombs on both sides and the matrix honeycomb the matrix honeycomb in the middle are subjected to severe compression, leading to the evolution of the original X-shaped deformation bands into three distinct I-shaped deformation bands. Upon reaching a strain of 0.72, the modular honeycomb transitions into a densified stage, making it challenging to discern the regular hexagonal cells.

**Figure 9:**
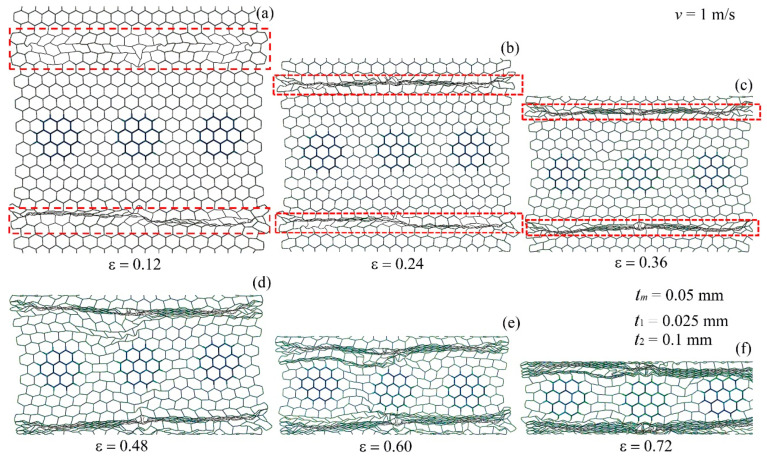
The deformation mode of the modular honeycomb when subjected to *x*-direction compression at the velocity of 1 m/s. (**a**) ε=0.12, (**b**) ε=0.24, (**c**) ε=0.36, (**d**) ε=0.48, (**e**) ε=0.60, (**f**) ε=0.72.

Figure 9 displays the deformation mode of the modular honeycomb during *x*-direction compression at a velocity of 1 m/s. Upon attaining a strain of 0.12, the sub-honeycombs with thin walls, characterized by reduced strength, lead to two plastic deformation zones (as indicated by the dashed lines) at the locations of the thin-walled sub-honeycombs. At a strain of 0.24, the thin-walled sub-honeycombs become fully dense, resulting in two I-shaped dense bands. Subsequently, the collapse of these dense bands progresses gradually from both ends toward the center, establishing a stable and orderly deformation mode. When the strain reaches approximately 0.6, most of the modular honeycomb transitions into the densified phase, concurrent with the initiation of deformation in the thick-walled sub-honeycombs. At a strain of 0.72, the thick-walled sub-honeycombs show slight deformations attributable to their enhanced strength, even though other layers of the modular honeycomb become densified.

The deformation mode of the modular honeycomb is characterized by initial plastic deformation and collapse of the thin-walled sub-honeycombs, preceding the progressive collapse in other matrix honeycombs when subjected to a 1 m/s compression along the *x*-direction. Subsequently, the thick-walled sub-honeycombs begin to deform. This sequential deformation pattern yields a stress–strain curve marked by several plateau regions for the modular honeycomb, which facilitates the targeted design of its mechanical response.

#### 4.1.2. The Dynamic Crushing Strength under Low-Velocity Crushing

The plateau stress of the modular honeycomb is an important reference of the structural resistance to load and the capacity for energy absorption, essential for assessing its resilience against impact. This section focuses on explaining the mechanism behind the mechanical reinforcement of the modular honeycomb when subjected to low-velocity impacts, by constructing a theoretical model for plateau stress. The plateau stress refers to the average stress experienced between the initial strain and the strain at densification.

**Figure 10:**
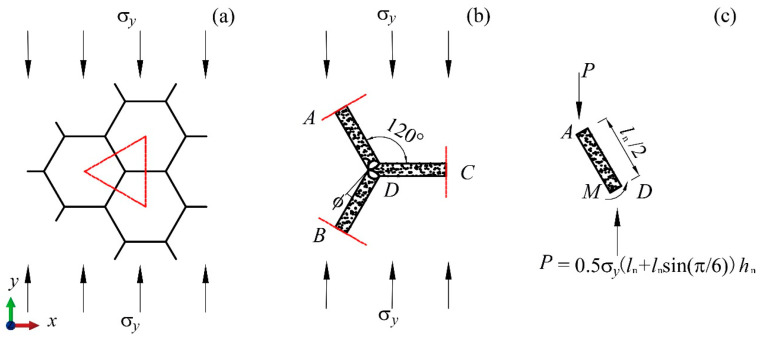
Analysis of force distribution in sub-honeycomb under compression along the *y*-axis. (**a**) A part of the sub-honeycomb, (**b**) the basic unit, (**c**) the strut AD.

Figure 10a presents the free-body diagram for a part of the sub-honeycomb during compression in the *y*-direction, while Figure 10b delineates the free-body diagram of the basic unit, highlighted by the red triangular area in Figure 10a. Figure 10c illustrates the force condition along strut AD. From the equilibrium of forces, it is deduced that the expression for the equivalent external force exerted on AD is as follows:

(7) $$P=0.5\sigma_{y}\left(l_{n}+l_{n}\sin\frac{\pi }{6}\right)h_{n}$$

During compression along the *y*-direction, both members AD and BD form plastic hinges at point D. The plastic hinges rotate 2π/3 when the honeycomb is dense, the energy dissipated through plastic hinges is:

(8) $$E_{p}=2M_{P}\frac{\pi }{3}$$

where $M_{P}$ can be obtained from the following equation:

(9) $$M_{P}=\frac{\sigma_{ys}h_{n}t_{n}^{2}}{4}$$

where $\sigma_{ys}$ denotes the stress at which the material begins to plastically deform.

For a honeycomb block with the layout BJ2, any sub-honeycomb contains 24 basic units. The total plastic energy absorbed upon complete deformation is

(10) $$E_{pn}=4\pi \sigma_{ys}h_{n}t_{n}^{2}$$

During low-velocity impacts, the matrix honeycomb experiences plastic energy dissipation in a similar manner to the sub-honeycomb. With a total of 648 basic units within the matrix honeycomb, the total plastic energy absorbed is calculated as follows:

(11) $$E_{pm}=108\pi \sigma_{ys}h_{m}t_{m}^{2}$$

The kinetic energy of the system is neglected under the low-velocity impact, with the assumption that the energy dissipated by plastic hinges is equal to the work exerted by external forces upon the honeycomb. Therefore,

(12) $$E_{pm}+\sum_{n=1}^{9}E_{pn}=Fs$$

where $F=36l_{m}h_{m}\sigma_{y}$ refers to the external forces; $s=36l_{m}\cos\left(\pi /6\right)$ represents the displacement over which the external force performs work.

Substituting the expressions of force *F* and displacement *s* into Equation (12) yields

(13) $$\sigma_{y}=\frac{E_{pm}+\sum_{n=1}^{9}E_{pn}}{648\sqrt{3}l_{m}^{2}h_{m}}$$

**Figure 11:**
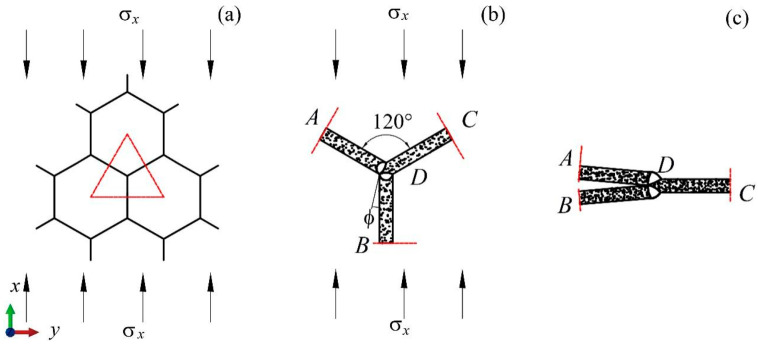
Analysis of force distribution in sub-honeycomb under compression along the *x*-axis. (**a**) A part of the sub-honeycomb, (**b**) the basic unit, (**c**) the deformation of the basic unit.

Figure 11a displays the free-body diagram of a sub-honeycomb under compression along the *x*-direction. Figure 11b provides a detailed force analysis of the basic unit, indicated by the red triangular area in Figure 11a. Figure 11c illustrates the densified form of the basic unit. The diagrams demonstrate that during *x*-direction compression, the total angle of rotation by the plastic hinges of the basic unit is 2π/3 too. Drawing from the earlier derivation of the average stress in the *y*-direction, the theoretical average stress for the BJ2 layout modular honeycomb during *x*-direction compression is formulated as follows:

(14) $$\sigma_{x}=\frac{E_{pm}+\sum_{n=1}^{9}E_{pn}}{1296{\varepsilon_{d}l}_{m}^{2}h_{m}}$$

#### 4.1.3. Generalized Scenario

In an extended application of a modular honeycomb, where the new design arranged with $N_{x}$ blocks along the *x*-direction and $N_{y}$ blocks along the *y*-direction, and each unit block contains $n_{x}$ cells in the horizontal direction and $n_{y}$ cells in the vertical direction, the BJ2 configuration of the block layout dictates the formulas for calculating the average stresses in the *y*- and *x*-directions. These stresses are denoted as $\sigma_{Ny}$ and $\sigma_{Nx}$, respectively, and the formulas are delineated as follows:

(15) $$\sigma_{yN}=\frac{{\sqrt{3}N}_{x}N_{y}\left(2n_{x}n_{y}-12\right)\pi \sigma_{ys}h_{m}t_{m}^{2}+12\sqrt{3}\sum_{n=1}^{N_{x}N_{y}}\pi \sigma_{ys}h_{n}t_{n}^{2}}{27N_{x}N_{y}n_{x}n_{y}{\varepsilon_{d}l}_{n}^{2}h_{m}}$$

(16) $$\sigma_{xN}=\frac{N_{x}N_{y}\left(n_{x}n_{y}-6\right)\pi \sigma_{ys}h_{m}t_{m}^{2}+6\sum_{n=1}^{N_{x}N_{y}}\pi \sigma_{ys}h_{n}t_{n}^{2}}{9N_{x}N_{y}n_{x}n_{y}{\varepsilon_{d}l}_{n}^{2}h_{m}}$$

To verify the reliability of Equations (15) and (16), the average stresses in the *x*- and *y*-directions of the modular honeycomb depicted in Figure 5 are calculated using numerical methods as well as the two formulas. In the numerical calculations, the formula for calculating the plateau stress is as follows:

(17) $$\sigma_{s}=\frac{\int_{\varepsilon_{cr}}^{\varepsilon_{d}}\sigma \left(\varepsilon \right)d\left(\varepsilon \right)}{\varepsilon_{d1}}$$

in which, $\sigma \left(\varepsilon \right)$ represents the stress, and $\varepsilon_{d1}$ is the strain at which the honeycomb’s energy absorption efficiency (EAE) reaches its last maximum value. The energy absorption efficiency is defined as the ratio of the honeycomb’s absorbed energy to the strain, that is,

(18) $$\varepsilon_{d1}=\frac{\int_{0}^{\varepsilon_{d}}\sigma \left(\varepsilon \right)d\left(\varepsilon \right)}{\sigma \left(\varepsilon \right)}$$

**Figure 12:**
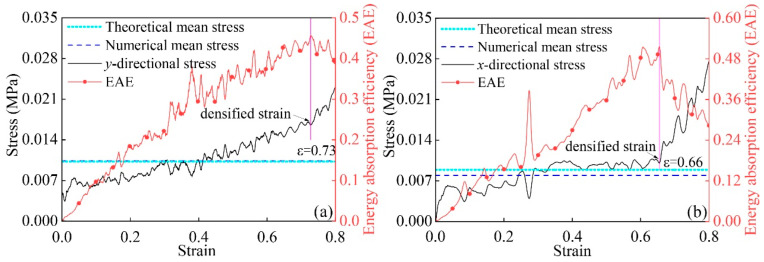
Stress–strain and energy absorption efficiency curves of the modular honeycomb under low-velocity impact: (**a**) *y*-direction; (**b**) *x*-direction.

Figure 12a illustrates the numerical stress and energy absorption efficiency for the structure under *y*-direction low-velocity impacts, complemented by the comparison of numerical and theoretical average stresses. Figure 12b corresponds with the results for low-velocity compression along the *x*-direction. The substantial agreement between the numerical and theoretical stresses validates the precision of the theoretical approach in forecasting the plateau stress for the modular honeycomb under quasi-static compression.

The plateau stress of the modular honeycomb under low-velocity impact correlates with several factors, including the quantity and arrangement of honeycomb blocks, the height of the sub-honeycomb and matrix honeycomb along the *z*-direction, the length of the cell walls in both the sub-honeycomb and matrix honeycomb, and the wall thickness of these components. Additionally, the yield strength of the basic material plays a crucial role. As a result, the average stress of modular honeycombs demonstrates tunability. In comparison to conventional honeycombs, the modular honeycomb cells display variability in the wall thicknesses. By examining Equations (15) and (16), it is evident that adjusting the wall thickness of modular honeycombs can yield a higher in-plane average stress than that of conventional honeycombs at identical relative densities. This accounts for the enhanced in-plane strength observed in modular honeycombs.

### 4.2. High-Velocity Crushing

#### 4.2.1. Deformation Mode under High-Velocity Crushing

**Figure 13:**
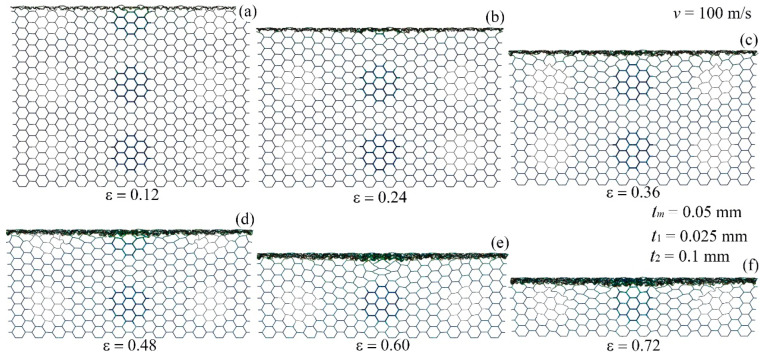
The deformation mode of the modular honeycomb when subjected to *y*-direction compression at the velocity of 100 m/s. (**a**) ε=0.12, (**b**) ε=0.24, (**c**) ε=0.36, (**d**) ε=0.48, (**e**) ε=0.60, (**f**) ε=0.72.

Figure 13 depicts the deformation of the modular honeycomb subjected to high-velocity compression (100 m/s) along the *y*-direction. The honeycomb demonstrates a consistent deformation pattern, marked by the emergence of I-shaped collapse bands at the end of the impact. These bands extend progressively in the direction of compression. At strains of 0.12, 0.24, 0.36, 0.48, 0.60, and 0.72, deformation is concentrated at the impact end, with the cells at the stationary end remaining undeformed. It is noteworthy that despite variations in wall thickness among the thin-walled sub-honeycombs, thick-walled sub-honeycombs, and the matrix honeycomb, there is no significant variation in the deformation mode under high-velocity impact.

**Figure 14:**
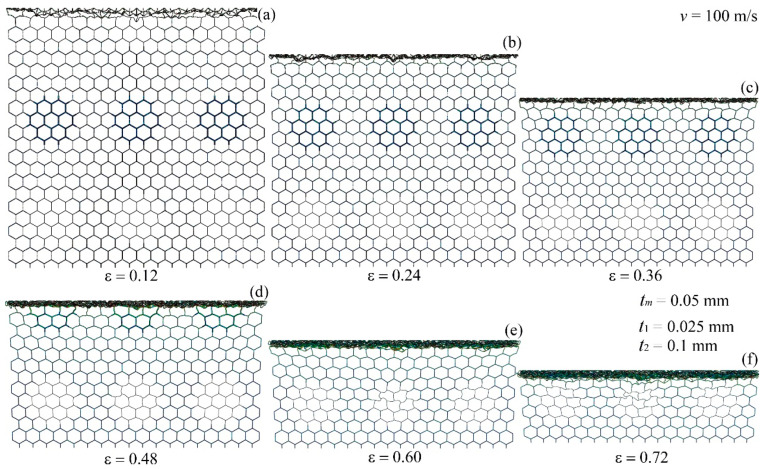
The deformation mode of the modular honeycomb when subjected to *x*-direction compression at the velocity of 100 m/s. (**a**) ε=0.12, (**b**) ε=0.24, (**c**) ε=0.36, (**d**) ε=0.48, (**e**) ε=0.60, (**f**) ε=0.72.

Figure 14 illustrates the deformation modes of the modular honeycomb when subjected to high-velocity compression along the *x*-direction at 100 m/s. At various strains, 0.12, 0.24, 0.36, 0.48, 0.60, and 0.72, the cells near the impact end densify, creating I-shaped deformation bands, whereas the cells at the stationary end show slight deformation. Figure 13 and Figure 14 show that the deformation modes under high-velocity compression in both *x*- and *y*-directions are analogous. The localized reinforcement of the honeycomb wall thickness exerts negligible influence on the deformation pattern. The deformation mode is predominantly influenced by the inertial effect under high-velocity impact.

#### 4.2.2. The Dynamic Crushing Strength under High-Velocity Crushing

The study [60] on conventional honeycombs shows that the cells at the impact end gradually densify towards the stationary end under high-velocity impacts, exhibiting a deformation mode similar to that of modular honeycombs. Reid et al. [61] and Zou et al. [62] established and refined the one-dimensional shock wave theory, and formulated expressions that capture the dynamic plateau stress observed within the conventional honeycomb in both the *x*- and *y*-directions. The expressions are as follows:

(19) $$\sigma_{d}=\sigma_{s}+Cv^{2}$$

in which, $\sigma_{d}$ and $\sigma_{s}$ represent the plateau stresses under dynamic and static compression, respectively. The term $Cv^{2}$ denotes the dynamic component, reflecting the influence of inertia and velocity on the dynamic plateau stress. v is the impact velocity, and C is the coefficient of the dynamic term, which can be estimated as follows:

(20) $$C=4742{\left(t/l\right)}^{2}+3115\left(t/l\right)+0.75$$

where t and l represent the wall thickness and edge length of the honeycomb, respectively.

**Figure 15:**
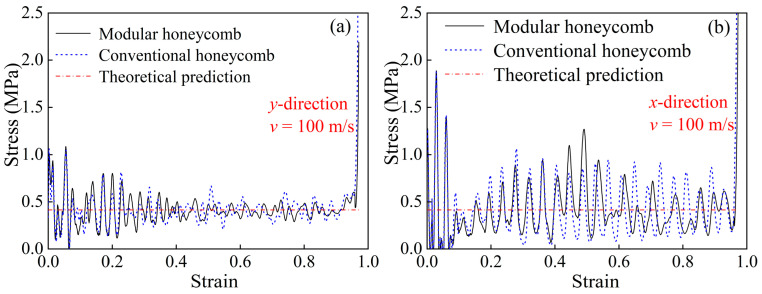
The stress–strain curves of the modular honeycomb and the conventional honeycomb under the impact velocity of 100 m/s, (**a**) *y*-direction, (**b**) *x*-direction.

High-velocity impact simulations are performed on the modular honeycomb shown in Figure 5, as well as on a conventional honeycomb with the same relative density, both subjected to an impact velocity of 100 m/s. The stress–strain curves under compression in the *y*- and *x*-directions are depicted in Figure 15a and Figure 15b, respectively. These curves for both honeycombs, regardless of the compression direction, display a notable similarity, suggesting that the inertial effect, equivalent density, and impact velocity are determinants of the plateau stress during high-velocity impacts. The dynamic compression theoretical formulas, derived from the conventional honeycomb, can be utilized to predict the plateau stress of the modular honeycomb.

To further validate the reliability of the theoretical formulas for the plateau stress of modular honeycombs under high-velocity impact, the relative density of the modular honeycomb, as depicted in Figure 5, is set to 3.46%. By adjusting the wall thicknesses of the sub-honeycombs and the matrix honeycomb, five distinct honeycombs are obtained as shown in Table 2. In group A, the wall thickness of the matrix honeycomb remains constant, while in group B, the wall thicknesses of both the sub-honeycombs and the matrix honeycomb vary. Group C serves as the control group with a conventional honeycomb, and the edge lengths of all honeycombs are 4 mm.

**Figure 16:**
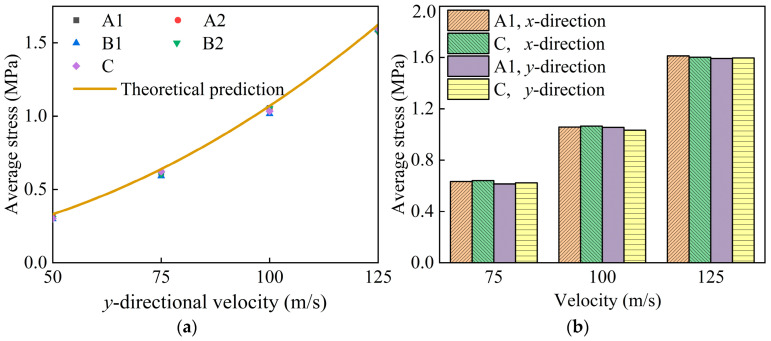
The plateau stresses for modular honeycombs and the conventional honeycomb under different impact velocities: (**a**) for impacts along the *y*-direction; (**b**) a comparative analysis along the *x*- and *y*-directions.

Figure 16a presents the plateau stress of honeycombs subjected to *y*-directional impacts at velocities ranging from 50 m/s to 125 m/s. Honeycombs with identical relative densities display comparable plateau stresses at a given velocity, aligning well with the theoretical predictions from Equation (19). Figure 16b compares the plateau stresses for honeycombs A1 and C under compression along the *x*- and *y*-directions at velocities of 75 m/s, 100 m/s, and 125 m/s. The bar chart underscores a negligible difference in plateau stress between the modular and conventional honeycombs of the same density. It also indicates that the variation in plateau stress for modular honeycombs under high-velocity impacts in both directions is slight. The study affirms the validity of employing the plateau stress formula for conventional honeycombs under high-velocity impacts to estimate the plateau stress of modular honeycombs under similar conditions.

## 5. Discussion

### 5.1. Densification Strain

**Figure 17:**
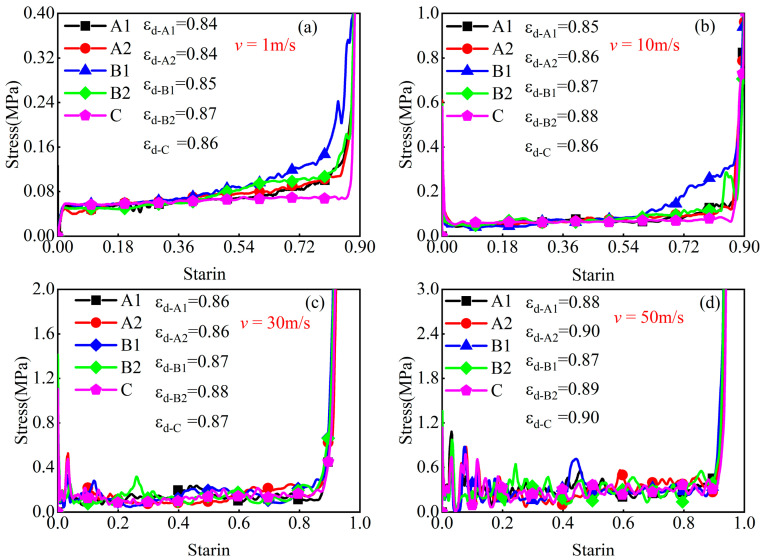
The stress–strain and densification strain of each honeycomb under varying velocities. (**a**) *v* = 1 m/s, (**b**) *v* = 10 m/s, (**c**) *v* = 30 m/s, (**d**) *v* = 50 m/s.

When a porous structure is subjected to compression and enters the densification phase, the internal pores undergo a closure process, leading to a sharp increase in stress and a significant decrease in the intended energy absorption capacity, thereby losing the protective capability for the object it intends to safeguard. To ensure that the modular honeycomb provides adequate energy absorption enhancement, its densification strain should be comparable to that of traditional honeycombs. The stress–strain curves of the four types of modular honeycombs and the corresponding conventional honeycombs presented in Table 2 are obtained using the numerical method, as illustrated in Figure 17. The densification strain of the structure is determined according to Equation (18), corresponding to the last peak of the curve. The results yielded the densification strain for the A1, A2, B1, B2, and C honeycombs under various impact velocities, as summarized in Table 3.

At an impact velocity of 1 m/s, the densification strains for these honeycomb structures are 0.84, 0.84, 0.85, 0.87, and 0.86, respectively. As the impact velocity increases, only slight variations in densification strain are noted. They are 0.85, 0.86, 0.87, 0.88, and 0.86 at 10 m/s. At 30 m/s, the values are 0.86, 0.86, 0.87, 0.88, and 0.87; and at 50 m/s, they are 0.88, 0.90, 0.87, 0.89, and 0.90. The data reveal that the maximum disparity in densification strain among the different modular honeycombs and the corresponding conventional honeycomb, all with the same relative density and tested across a spectrum of impact velocities, is a mere 3.33%. This finding implies that variations in the wall thickness of modular honeycombs exert a negligible influence on the densification strain.

### 5.2. Energy Absorption

**Figure 18:**
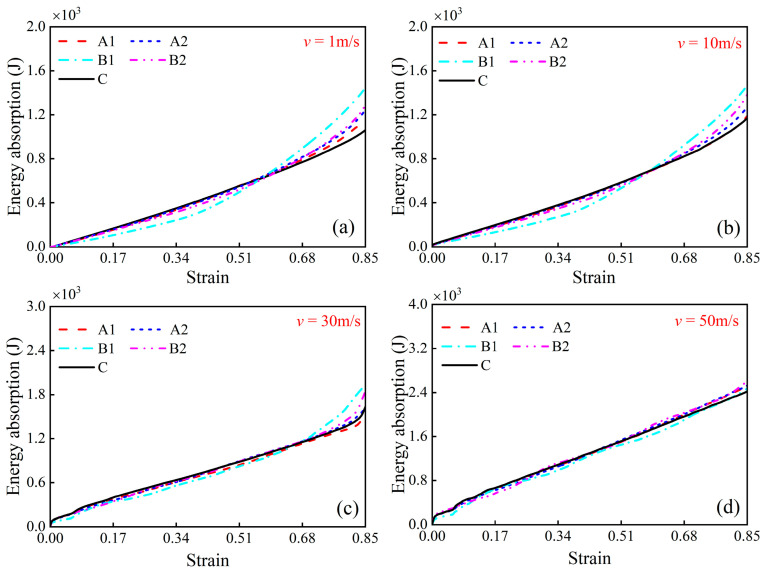
Energy absorption of various honeycombs with an equivalent density of 3.46% under different impact velocities. (**a**) *v* = 1 m/s, (**b**) *v* = 10 m/s, (**c**) *v* = 30 m/s, (**d**) *v* = 50 m/s.

The capacity for energy absorption is an essential attribute of honeycombs. Figure 18a–d sequentially plot the energy absorption curves for the honeycombs enumerated in Table 2 under impact velocities of 1 m/s, 10 m/s, 30 m/s, and 50 m/s, respectively. Given that the relative densities of all the honeycombs are uniform, the curves in Figure 18 can also represent the specific energy absorption trends characteristic of each honeycomb. A comparative analysis between Figure 17b and Figure 18a indicates that increasing the velocity from 1 m/s to 10 m/s leads to only a slight change in energy absorption for all honeycombs. The energy is primarily absorbed by the formation of plastic hinges within the honeycomb. As the velocity further increases to 30 m/s and 50 m/s, the energy absorption of each honeycomb increases markedly. The enhancement in inertial and strain rate effects under high-velocity impact is the main reason for the increased energy absorption.

Additionally, Figure 18a,b demonstrate that the energy absorption of the modular honeycombs is initially less than that of the conventional honeycomb under low-velocity impact. However, once the strain exceeds about 0.6, the modular honeycombs gradually surpass the conventional honeycomb in energy absorption. The deformation modes of modular honeycombs under low-velocity compression can elucidate this phenomenon. At the initiation of compression in the modular honeycomb, the internal thin-walled sub-honeycombs are the primary structures that undergo deformation, resulting in lower energy absorption compared to the conventional honeycomb at equivalent strain. As compression progresses, the thick-walled sub-honeycombs within the modular structure take over as the main contributors to deformation, resulting in a greater energy absorption compared to the conventional honeycomb. At a strain of 0.85, the energy absorption capacity of the B1 honeycomb increases by 36.68% and 25.47% compared to the C honeycomb at velocities of 1 m/s and 10 m/s, respectively, marking a significant enhancement. The energy absorption of the modular honeycomb ultimately exceeds that of the conventional honeycomb, indicating that the modular honeycomb can enhance the energy absorption capacity under low-velocity compression. Figure 18c,d show that the energy absorption of all honeycombs at an impact velocity of 30 m/s and 50 m/s is nearly identical. The results imply that adjustments in the local wall thickness of modular honeycombs have a negligible impact on the energy absorption capacity of the structure under high-velocity impact.

### 5.3. Customized Strategies for Uniaxial Compression

**Figure 19:**
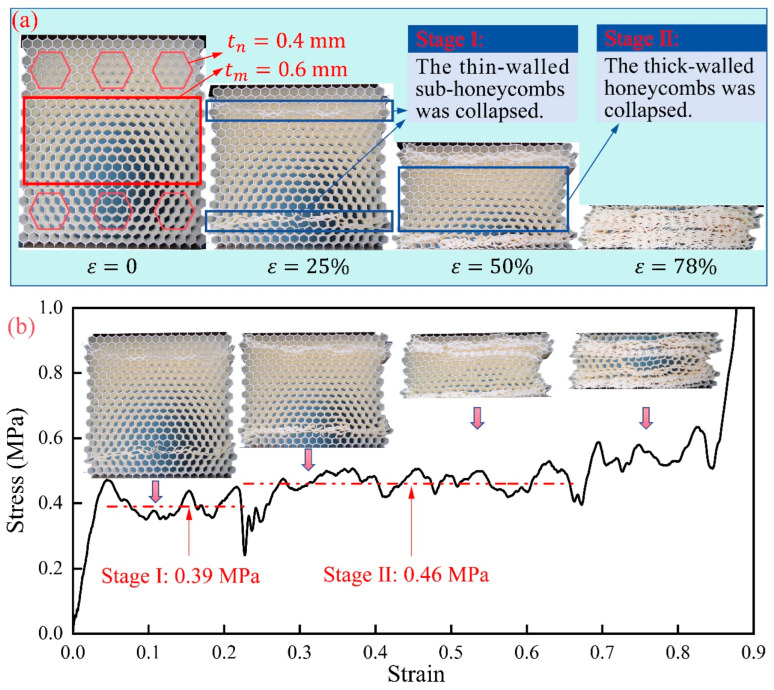
Customized response by intermixing two types of honeycombs with different cell walls. (**a**) Deformation process; and (**b**) stress versus strain curve.

Figure 19a illustrates the deformation modes of a modular honeycomb fabricated via additive manufacturing under uniaxial compression. The modular honeycomb was fabricated from PLA material via additive manufacturing on a Raise3D Pro2 Plus (Shanghai Fuzhi Information Technology Co., Ltd., Shanghai, China) printing platform, with a nozzle temperature set at 205 °C, a platform temperature of 60 °C, and a nozzle diameter of 0.4 mm. The material’s elastic modulus and yield strength are characterized as 2.1 GPa and 39.8 MPa, respectively. It comprises six thin-walled sub-honeycombs with a wall thickness of 0.4 mm, while the remainder of the structure consists of cells with a uniform wall thickness of 0.6 mm. It demonstrates that the compressive deformation occurred in two phases. Initially, the thin-walled sub-honeycombs underwent collapse, followed by the collapse of the thicker-walled honeycomb in the second phase.

The stress–strain curve also exhibits two-stage plateau stress in Figure 19b. The first plateau stress corresponds to the compression deformation of the thin-walled sub-honeycombs, with a stress of 0.39 MPa, while the second plateau corresponds to the deformation phase of the thicker-walled honeycomb, with a stress of 0.46 MPa. Subsequently, the compression process transitions into a densification phase. The phenomena can be attributed to the low relative density and correspondingly lower load-bearing capacity of the thin-walled sub-honeycombs, which makes them more susceptible to collapse. Drawing inspiration from this mechanism, it is possible to tailor a modular honeycomb with multi-stage mechanical response and determine their respective deformation modes by adjusting the layout and the wall thickness of sub-honeycombs.

**Figure 20:**
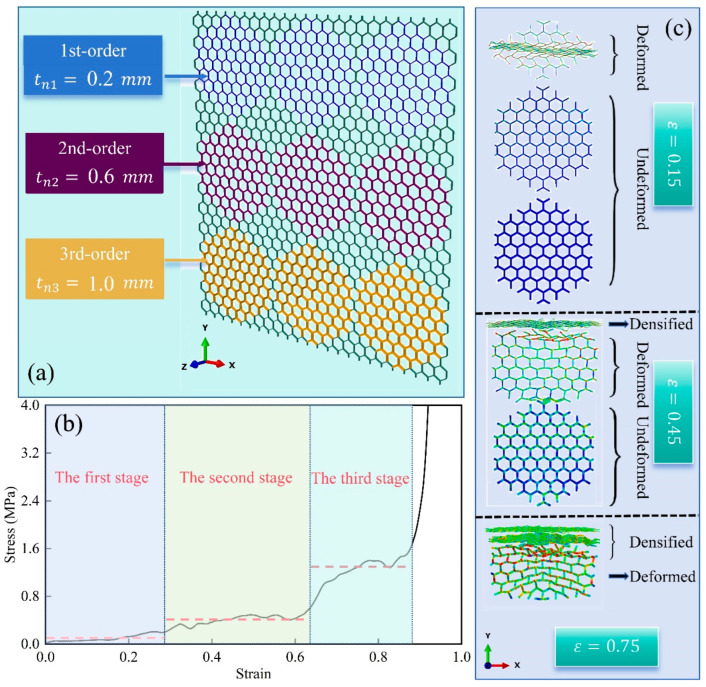
Design of a modular honeycomb with three-stage plateau stress: (**a**) geometric model, (**b**) stress–strain curve, (**c**) deformation process.

For instance, to meet the needs of engineering applications that require an energy absorber with a three-stage stress plateau, Figure 20a presents a customized modular honeycomb fabricated by PLA. This design features sub-honeycombs, categorized as 1st-order, 2nd-order, and 3rd-order, each with respective cell wall thicknesses of 0.2 mm, 0.6 mm, and 1.0 mm. These are strategically arranged in increasing order of relative density, with the rows sequenced from the top to the bottom. Figure 20b displays the stress–strain curve of the modular honeycomb under in-plane compression, characterized by three distinct and sequentially higher plateau stresses. Figure 20c illustrates the deformation of sub-honeycombs at strains of 0.15, 0.45, and 0.75, respectively. At a strain of 0.15, sub-honeycombs with a wall thickness of 0.2 mm exhibit plastic deformation, while those with thicker walls do not undergo plastic deformation, corresponding to the first plateau of the stress–strain curve. As the strain increases to 0.45, the sub-honeycombs with a 0.2 mm wall thickness are densified, and those with a 0.6 mm wall thickness start to plastically deform, while the 1.0 mm walled sub-honeycombs remain undeformed, corresponding to the second plateau. Upon reaching a strain of 0.75, sub-honeycombs with wall thicknesses of 0.2 mm and 0.6 mm are desertified, and the 1.0 mm walled sub-honeycombs commence deformation, corresponding to the third plateau stress. The deformation process of the modular honeycomb and the number of stress–strain curve plateaus align with expectations, confirming the feasibility of achieving multi-stage stress design by adjusting the arrangement and wall thickness of sub-honeycombs. This design approach provides an effective solution for the intelligent customization of responses in engineering applications.

### 5.4. Customized Strategies for Biaxial Compression

**Figure 21:**
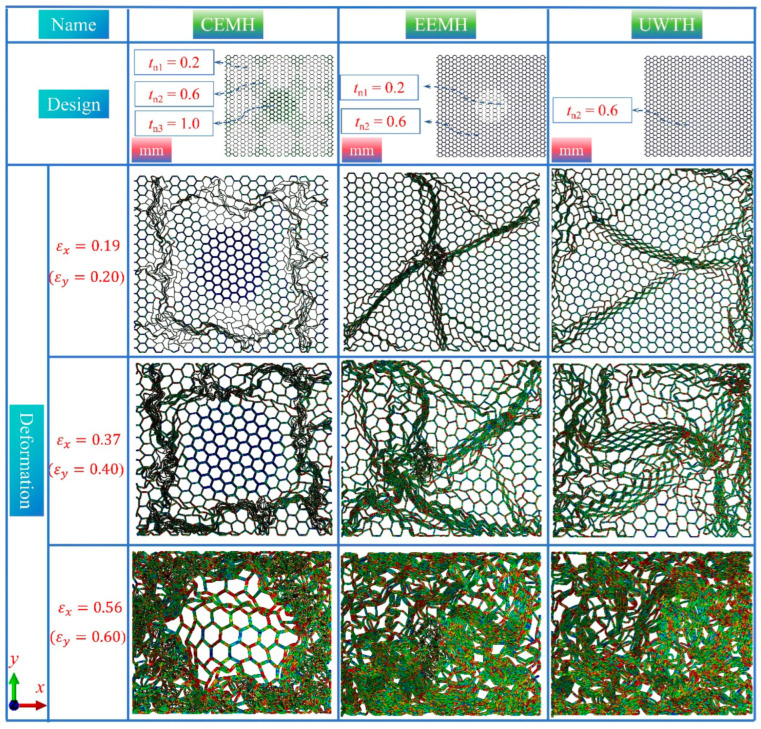
The geometric configurations of the central enhanced modular honeycomb (CEMH), external enhanced modular honeycomb (EEMH), and uniform wall thickness honeycomb (UWTH), as well as the deformation modes of these three types of honeycombs under biaxial compression at longitudinal strains of 0.2, 0.4, and 0.6.

This section introduces the design of two distinct types of modular honeycombs, aimed at demonstrating the potential for customization in their response to biaxial compression. The first variant is the central enhanced modular honeycomb (CEMH), which features a central sub-honeycomb wall measuring 1.0 mm in thickness, surrounded by thinner sub-honeycomb walls at 0.2 mm, and a matrix honeycomb wall thickness of 0.6 mm. The second variant, the external enhanced modular honeycomb (EEMH), inverts this design, with a central sub-honeycomb wall of 0.2 mm thickness complemented by matrix and other sub-honeycomb walls at 0.6 mm. For comparative analysis, a uniform wall thickness honeycomb (UWTH) with a consistent 0.6 mm wall thickness across all components is also presented, as illustrated in Figure 21. The dimensions of these honeycombs in the *x*- and *y*-axes are 180.0 mm and 166.3 mm, respectively. Figure 21 presents a comparative study of the deformation modes of these honeycombs under biaxial compression at longitudinal strains of 0.2, 0.4, and 0.6. At a strain of 0.2, the CEMH exhibits significant plastic deformation in the thinner sub-honeycomb walls, while the central region with 1.0 mm wall thickness remains largely undeformed. Conversely, the central sub-honeycomb compacts in the EEMH, creating distinct compaction bands radiating outward. The UWTH displays a disorder deformation mode. As the strain increases to 0.4, the thinner sub-honeycombs in the CEMH are fully compacted, and the matrix honeycomb is severely deformed, yet the central sub-honeycombs with 1.0 mm wall thickness remain largely intact. The EEMH shows more pronounced compaction bands, while the UWTH becomes increasingly disordered. At the strain of 0.6, the central sub-honeycombs in the CEMH, despite not being fully compacted, are surrounded by a fully dense matrix and sub-honeycombs. Both the EEMH and UWTH achieve a state of complete compaction.

Collectively, these observations suggest that the CEMH and EEMH offer a higher degree of control over deformation modes compared to the UWTH. This control is indicative of the ability to tailor the deformation characteristics of modular honeycombs under biaxial compression by adjusting the wall thicknesses of sub-honeycombs and the matrix honeycomb, thereby enabling a customized approach to structural design.

**Figure 22:**
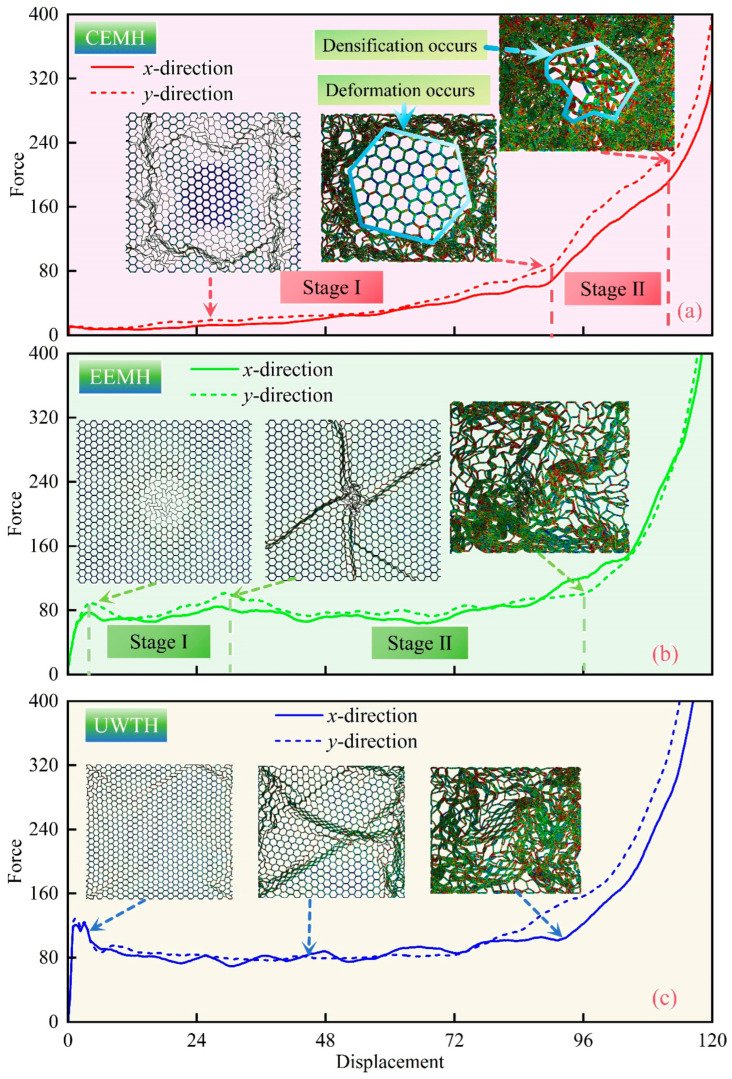
The *x*- and *y*-directional force–displacement curves of (**a**) CEMH, (**b**) EEMH, and (**c**) UWTH under biaxial compression.

Figure 22 delineates the force–displacement curves for the CEMH, EEMH, and UWTH when subjected to biaxial compression. Figure 22a illustrates that the CEMH progresses through two distinct phases before densification. Initially, there is a gradual increase in force, indicative of the initial compression phase involving the thinner sub-honeycombs and the matrix honeycomb. Subsequently, the second phase is initiated by the deformation of the thicker sub-honeycombs, leading up to the point of densification. This bipartite behavior of the CEMH’s force–displacement curve is ascribed to the substantial wall thickness of the thicker sub-honeycombs, which provides them with a superior load-bearing capability compared to their thinner counterparts and the matrix honeycomb.

Figure 22b elucidates that the EEMH’s force–displacement curves also exhibit a two-stage plateau phase. The curve graph highlights two significant peaks that correspond to the onset of the deformation and densification of the thin-walled sub-honeycombs, respectively. It is observable that the minimal variation in force occurs across the two phases, attributable to the central thin-walled sub-honeycombs having a wall thickness of merely 0.2 mm, which minimally affects the load-bearing capacity of the EEMH during the deformation process. Figure 22c demonstrates that under biaxial compression, the UWTH’s force–displacement curves in both the *x*- and *y*-directions exhibit a singular plateau phase, which is not markedly different from that observed under uniaxial compression.

A comparative analysis of the force–displacement curves among the three honeycomb types indicates that by strategically adjusting the wall thickness and arrangement of the sub-honeycombs within the modular honeycomb, it is feasible to achieve a segmented modulation of the force–displacement response in both directions. Such tuning holds considerable practical significance in engineering applications that necessitate precise control over the structural behavior under biaxial compression, such as in the logistics industry where packaging materials are designed to absorb and distribute impact forces from various directions to safeguard the contents. By refining the honeycomb structure design, the protective efficiency of packaging materials and the structural load-bearing performance can be optimized, thereby facilitating a more precise and targeted engineering approach.

## 6. Conclusions

In this study, a novel biomimetic modular honeycomb design is introduced, aimed at enhancing in-plane compressive strength and customizability of plateau stress. Through detailed analysis and experiment, major conclusions can be drawn as follows:

1. Biomimetic Design: Drawing inspiration from the natural density variation observed in tree trunks, this work effectively integrates this concept into the honeycomb structure design. The novel honeycomb features a bio-inspired modular design, where the walls of the sub-honeycombs in a conventional honeycomb are selectively thickened. Utilizing the formula for calculating the relative density of porous structures, a method for calculating the relative density of modular honeycombs is provided.
2. Reinforcement Mechanisms: Across a range of impact velocities, the densification strain in modular honeycombs remains comparable to that of traditional honeycombs with similar relative densities. Nonetheless, increasing the cell wall thickness augments the plateau stress of modular honeycombs during low-velocity impacts, thereby enhancing their energy absorption capability. At a strain of 0.85, the energy absorption capacity of modular honeycomb can be enhanced by up to 36.68% and 25.47% compared to conventional honeycomb at velocities of 1 m/s and 10 m/s, respectively. Nearly identical densification strains coupled with increased plateau stresses constitute the principal mechanism that supports the superior energy absorption capabilities of modular honeycombs.
3. Flexible customizability: Under low-velocity impacts, modular honeycombs display distinctive deformation modes, which are modifiable through adjustments in the sub-honeycomb configurations, making them different from traditional honeycombs. Conversely, at high velocities, the deformation mode in modular honeycombs mirrors that of traditional ones, marked by a densification that propagates from the impact end to the fixed end. Inspired by the deformation mechanisms observed during low-velocity impacts, it is possible and practical to design a modular honeycomb that exhibits a multi-stage mechanical response. This can be achieved by meticulously adjusting the arrangement and wall thickness of the sub-honeycombs, thereby devising their distinct deformation modes under both uniaxial and biaxial compression.

In conclusion, the innovative biomimetic modular honeycomb design presented in this study demonstrates a significant advancement in the field of cellular materials. The design not only bolsters the in-plane compressive strength through strategic wall thickening but also introduces customizability in plateau stress, catering to diverse application requirements, contributing valuable insights into the design of the conventional honeycomb, and setting a solid foundation for future work in the field, promising incremental yet meaningful improvements in material science and related engineering disciplines.

## Figures and Tables

**Table 1:** Plastic hardening parameters for the base material in the numerical model [58].

Strain	Stress (MPa)	Strain	Stress (MPa)
0	80.09	0.094	174.01
0.024	117.95	0.116	188.12
0.048	146.09	0.138	196.91
0.071	161.43	0.160	203.54

**Table 2:** The wall thicknesses of five types of honeycombs.

Group	Types	Sub-Honeycomb a (mm)	Sub-Honeycomb b (mm)	Matrix Honeycomb (mm)	Relative Density
A	A1	0.09	0.18	0.12	3.46%
	A2	0.15	0.06	0.12	3.46%
B	B1	0.18	0.27	0.09	3.46%
	B2	0.05	0.08	0.14	3.46%
C	C1	0.12	0.12	0.12	3.46%

**Table 3:** The densification strain of each honeycomb under varying impact velocities.

	Velocity	1 m/s	10 m/s	30 m/s	50 m/s
Type					
A1		0.84	0.85	0.86	0.88
A2		0.84	0.86	0.86	0.90
B1		0.85	0.87	0.87	0.87
B2		0.87	0.88	0.88	0.89
C		0.86	0.86	0.87	0.90
Maximum difference		2.33%	2.33%	1.15%	3.33%

## Data Availability

The original contributions presented in the study are included in the article, further inquiries can be directed to the corresponding author.
